# Effect of prenatal screening on trends in perinatal mortality associated with congenital anomalies before and after the introduction of prenatal screening: A population‐based study in the Northern Netherlands

**DOI:** 10.1111/ppe.12792

**Published:** 2021-07-30

**Authors:** Francesca Bardi, Jorieke E. H. Bergman, Katelijne Bouman, Jan Jaap Erwich, Leonie K. Duin, Hermien E. K. Walle, Marian K. Bakker

**Affiliations:** ^1^ Department of Obstetrics and Gynecology University Medical Centre Groningen Groningen The Netherlands; ^2^ Department of Genetics EUROCAT Registration Northern Netherlands University Medical Centre Groningen Groningen The Netherlands

**Keywords:** congenital anomalies, foetal mortality, perinatal mortality, prenatal screening

## Abstract

**Background:**

Perinatal mortality in foetuses/children with congenital anomalies remains high. Prenatal diagnosis, essential for risk assessment and organisation of perinatal/postnatal care, offers parents the opportunity to consider the termination of pregnancy. In times of quick changes in prenatal screening programmes, it is relevant to evaluate the effect of prenatal screening on perinatal mortality rates.

**Objectives:**

The objective of this study was to study trends in early foetal and perinatal mortality associated with congenital anomalies before/after the introduction of the Dutch prenatal screening programme.

**Methods:**

This population‐based cohort study included 8535 foetuses/neonates with congenital anomalies born in the Northern Netherlands between 2001 and 2017. Total deaths were defined as sum of early foetal (before 24 weeks’ gestation) and perinatal deaths (from 24 weeks’ gestation till day 7 post‐partum). Foetal deaths were categorised into spontaneous or elective termination of pregnancy for foetal anomalies (TOPFA). Trends in total mortality as well as early foetal and perinatal mortality were studied. Joinpoint regression was used to calculate the average annual percentage chance (AAPC) and identify linear trends in mortality within subperiods.

**Results:**

Total and perinatal mortality were 17% and 4%. Total mortality was higher in abnormal karyotype and central nervous system anomalies. We observed an increase in total mortality over time: 11.9% in 2001 versus 21.9% in 2017 (AAPC 2.6, 95% confidence interval [CI] 1.5, 3.7), caused by an increase in early foetal mortality from 5.5% to 19.2% (AAPC 8.7, 95% CI 4.7, 12.9) and a decrease in perinatal mortality from 6.4% to 2.7% (AAPC −5.6, 95% CI −10.0, −1.0). The increase in early foetal mortality reflects an increase in TOPFA from 3.6% to 16.9% (AAPC 8.3, 95% CI 4.2, 12.7), mostly occurring at 13–14 and 20–23 weeks’ gestation.

**Conclusions:**

The introduction of the prenatal screening programme led to a decrease in perinatal mortality among foetuses and neonates with congenital anomalies and a marked increase in early foetal mortality before 24 weeks’ gestation due to higher rates of TOPFA.

**Synopsis**

1 **Study question**

Does the introduction of prenatal screening affect foetal and perinatal mortality rates in foetuses and neonates with congenital anomalies?

2 **What is already known**

Perinatal mortality rates in foetuses and children with congenital anomalies are high. Prenatal screening leads to earlier diagnosis of congenital anomalies during pregnancy.

3 **What this study adds**

The introduction of prenatal screening causes a clear shift from late foetal and neonatal mortality to early foetal mortality and a notable difference in the percentage of foetal and neonatal deaths. A much larger number of terminations of pregnancy for foetal anomalies are performed at lower gestational ages. Foetal mortality rates peak at 13–14 and at 21–23 weeks of gestation, in concomitance with the first‐trimester screening and the second‐trimester anomaly scan.

## 1 BACKGROUND

Congenital anomalies account for ~20% of foetal deaths in Europe and are the main contributor to perinatal death in high‐income countries.^1^, ^2^ For this reason, and given their high psychological and financial burden on individuals, families, healthcare systems and society, congenital anomalies are an important focus of public health. Congenital anomalies are mostly caused by (a combination of) demographic, genetic or environmental factors, but the aetiology remains unknown in about half of cases.^2^, ^3^ Early diagnosis during pregnancy is therefore essential for risk assessment and organisation of perinatal and postnatal care.

In the Netherlands, pregnant women are offered an early scan at about 10 weeks of gestation to establish pregnancy viability, multiplicity/chronicity and to correctly date the pregnancy. At this early stage, foetal anatomical assessment does not take place. In 2007, a nationwide prenatal screening programme was implemented in the Netherlands.^4^ This programme included the combined test (CT) for trisomies 21, 13 and 18 and the 20‐week anomaly scan for structural anomalies. The 20‐week scan is performed following a systematic protocol for foetal anatomical assessment by qualified sonographers who meet the required quality standards.^5^ In 2014, non‐invasive prenatal testing (NIPT) using cell‐free foetal DNA became available to women showing increased risk on the CT or with a previous child with a trisomy. From April 2017, NIPT was offered to all women as first‐tier screening in the first trimester of pregnancy. However, while the 20‐week anomaly scan is covered by mandatory health insurance, the woman has to pay around 170 euro for the CT and NIPT. In the Northern Netherlands, the uptake of the 20‐week scan (82%) has consistently been higher than the CT (32%) or NIPT (29%).^6^ The implementation of and subsequent changes to the national prenatal screening programme have led to an increase in prenatal detection of several kinds of congenital anomalies, including neural tube defects, heart defects and urinary tract anomalies.^7^, ^8^, ^9^, ^10^ Earlier diagnosis offers the opportunity of timely and appropriate counselling to parents on the prognosis of their child and the various therapeutic options available. This allows parents more time to make an informed decision on the management or continuation of the pregnancy and healthcare professionals to properly organise postnatal care. While most studies have focused on prenatal detection rates of congenital anomalies, data on trends in mortality rates following prenatal screening introduction are less frequently reported in the literature.

Because our hypothesis is that these important changes in the prenatal screening programme are likely to reshape mortality rates, we set out to study trends in early foetal and perinatal mortality associated with congenital anomalies in the Northern Netherlands over the time period in which the national prenatal screening programme was implemented. Secondly, we aimed to study trends in foetal and perinatal mortality associated with the different types of foetal congenital anomalies.

## 2 METHODS

### 2.1 Study design and setting

This is a population‐based cohort study including 8535 foetuses and neonates with congenital anomalies born in the Northern Netherlands between 2001 and 2017. Data were extracted from Eurocat Northern Netherlands (Eurocat NNL), a population‐based birth defects registry covering the three Northern provinces of the Netherlands, which together account for approximately 10% of births in the Netherlands yearly. Eurocat NNL cases are identified by active case ascertainment using multiple sources, including hospital files, prenatal diagnosis reports and post‐mortem examinations. There is no gestational age limit for registration. Terminations of pregnancy due to foetal anomalies (TOPFAs), allowed until 24 weeks of gestation in the Netherlands, and spontaneous foetal deaths are therefore included in the data. The upper age limit for inclusion is 10 years.

Cases are registered in the database after parental consent. For pregnancies delivered/terminated from 2010 onwards, basic information was registered (anomalies type, way of delivery, time of diagnosis) when parents did not respond after a second request for consent. Information on these so‐called ‘non‐responders’ is registered to enable accurate monitoring of the occurrence of congenital anomalies. For each child/foetus, information is recorded on specific congenital anomalies, overall diagnosis, year of birth, pregnancy outcome (live birth, stillbirth, TOPFA, spontaneous foetal death), date of death (if occurred postnatally), gestational age (in weeks) at birth or at TOPFA and time of detection of congenital anomalies (prenatal, at birth, post‐partum). If the anomaly was detected prenatally, information on prenatal tests and gestational age at diagnosis was recorded. All congenital anomalies in the database are coded by trained registry staff following EUROCAT guide 1.4 using the ICD10‐BPA classification system (International Classification of Diseases version 10 with the British Paediatric Association extension).^11^, ^12^

### 2.2 Cohort

We included all foetuses and neonates with major congenital anomalies born between 2001 and 2017. Cases were categorised according to congenital anomalies the following subgroups: isolated structural anomalies, multiple congenital anomalies (MCA), abnormal karyotype and other syndromes. Isolated structural anomalies were further subdivided into anomalies of the following systems: central nervous, cardiac and circulatory, digestive, urogenital, musculoskeletal/other organs and orofacial clefts. MCA was defined as two or more unrelated congenital anomalies in more than one organ system. The abnormal karyotype group included trisomies 13, 18 and 21, monosomy X and triploidy detectable by QF‐PCR. Other syndromes included teratogenic and genetic syndromes and associations.

For cases registered with parental consent, we included information on maternal and pregnancy characteristics. Because maternal age ≥36 years was previously an indication for direct invasive diagnostic testing or CT free of charge, we divided maternal age into two categories: <36 years and ≥36 years. Ethnicity was defined as Western and non‐Western based on the country of birth of the maternal grandparents. Educational level was classified as low (primary school, lower vocational and prevocational education), middle (secondary vocational education, general secondary education and pre‐university education) and high (college or university education) based on the self‐reported highest educational level achieved. Plurality was defined as singleton and multiple births and gravidity as primigravida and multigravida.

### 2.3 Outcomes

For all cases, we defined pregnancy outcomes. We used the Dutch gestational age limit for termination of pregnancy (24 weeks) in the definitions of foetal, neonatal and perinatal mortality (see Table 1). In addition, we categorised foetal mortality according to whether death occurred by elective TOPFA or spontaneously. Induced delivery after foetal demise was categorised as spontaneous foetal death.

**TABLE 1:** Definitions of mortality used in this study

Early foetal mortality	Foetal death (spontaneous and elective) and stillbirths before 24 completed weeks of gestation
Late foetal mortality	Foetal death (spontaneous and elective) and stillbirths at or after 24 completed weeks of gestation
Total foetal mortality	Combined early and late foetal mortality
Early neonatal mortality	Death in live births (at or after 24 completed weeks of gestation) until day 7 after birth
Perinatal mortality	Combined late foetal and early neonatal mortality (at or after 24 completed weeks of gestation till day 7 after birth)
Total mortality	Combined early foetal mortality and perinatal mortality

### 2.4 Statistical analysis

We examined trends in mortality among foetuses with congenital anomalies between 2001 and 2017 for total mortality, perinatal mortality and early foetal mortality (TOPFA/spontaneous). Since the legal gestational age limit for TOPFA in the Netherlands is 24 weeks, we examined trends in mortality using 24 weeks as the cut‐off point.

We used joinpoint regression analysis to identify linear trends in mortality that are restricted to subperiods of varying sizes based on the presence of similar linear trends within each period. Joinpoint analyses calculate the annual percentage change (APC) in rates between years when a trend change is produced (joinpoints). If no joinpoints are identified, no trend changes within the study period are identified. The average annual percent change (AAPC) over the whole study period was calculated as a weighted average of the APCs from the joinpoint model, with the weights equal to the length of APC interval.

Analyses were performed in IBM SPSS Statistics for Windows, version 23.0.0.3 (Armonk, NY: IBM Corp.). Joinpoint regression analyses were performed using Joinpoint Trend Analysis Software (version 4.8.0.1) from the National Cancer Institute.^13^

### 2.5 Missing data

Data were complete for the core variables used to study trends in mortality: congenital anomalies and overall diagnosis, year of birth, pregnancy outcome, date of death, gestational age at birth or at TOPFA and (gestational age at) time of detection of the congenital anomaly. For maternal and pregnancy characteristics, the following data were missing: information on child's gender and maternal age at birth was missing in <1% of cases, information on pregnancy characteristics was missing in 6%–7% of cases and information on maternal ethnicity and educational level, mainly obtained from a parental questionnaire, was missing in approximately 20% of cases. Since we did not perform trend analyses in relation to these characteristics, we reported those characteristics without adjustments for missing data.

### 2.6 Ethics approval

This study was performed with anonymised data. Ethical approval by the Medical Ethical Committee was waived due to the retrospective nature of the study and because parental consent was obtained for data registration.

## 3 RESULTS

### 3.1 Mortality according to maternal and pregnancy characteristics

Between birth years 2001 and 2017, 8535 foetuses and neonates with congenital anomalies were registered in the Eurocat NNL database, corresponding to a prevalence of 2.84 per 100 births (95% CI 2.78, 2.91). Total mortality among these foetuses/children was 17% (*n* = 1489). Early foetal mortality was 13% (*n* = 1142), of which the majority were TOPFAs (*n* = 997; 12%). Perinatal mortality was 4% (*n* = 347), and about half of these were early neonatal deaths (*n* = 182; 2%).

When only including cases registered with parental consent (*n* = 7313; 86%), total mortality was higher in mothers aged 36 years and older, of non‐Western ethnicity, multigravidae and with female children. Mortality also increased with educational level, in particular early foetal mortality. Most deaths in singletons occurred before the legal limit for TOPFA (24 weeks of gestation), whereas deaths occurred equally before, at or after 24 weeks in pregnancies with multiple gestation (Table 2).

**TABLE 2:** Mortality among cases with congenital anomalies according to selected maternal, pregnancy and foetal characteristics (*n* = 7313); Eurocat Northern Netherlands, 2001–2017

	Total births	Total mortality (early foetal and perinatal mortality combined)	Early foetal mortality <24 weeks	Perinatal mortality ≥24weeks‒7 days after birth
	*N*	*n* (%)	*n* (%)	*n* (%)
Maternal age				
15–35	5992	921 (15.4)	677 (11.3)	244 (4.1)
≥36	1264	359 (28.4)	289 (22.9)	70 (5.5)
*Missing*	*57*			
Ethnicity				
Western	5753	1008 (17.5)	755 (13.1)	253 (4.4)
Non‐Western	302	66 (21.9)	46 (15.2)	20 (6.6)
*Missing*	*1258*			
Educational Level				
Low	711	88 (12.4)	54 (7.6)	*34 (4.8)*
Middle	2798	464 (16.6)	342 (12.2)	122 (4.4)
High	2394	487 (20.3)	388 (16.2)	99 (4.1)
*Missing*	*1410*			
Sex of child				
Male	4312	648 (15.0)	472 (10.9)	176 (4.1)
Female	2987	618 (20.7)	482 (16.1)	136 (4.6)
*Unknown*	*14*			
Plurality				
Singleton	6504	1195 (18.4)	918 (14.1)	277 (4.3)
Multiple births	340	63 (18.5)	30 (8.8)	33 (9.7)
*Missing*	*469*			
Gravidity				
1	2361	399 (16.9)	298 (12.6)	*101 (4.3)*
>=2	4404	849 (19.3)	644 (14.6)	205 (4.7)
*Missing*	*548*			

Note

The Grey Shade terms definitions are Total foetal mortality: Combined early and late foetal mortality, Early foetal mortality: Foetal death (spontaneous and elective) and stillbirths before 24 completed weeks of gestation, Perinatal mortality: Combined late foetal and early neonatal mortality (at or after 24 completed weeks of gestation till day 7 after birth).

### 3.2 Mortality according to type of congenital anomaly

The highest total mortality was among cases with abnormal karyotype and isolated anomalies of the central nervous system. For the abnormal karyotype cases, mortality was more frequent before 24 weeks’ gestation (54%), and the majority of these early foetal deaths were TOPFAs. Perinatal mortality was highest among cases with isolated anomalies of the central nervous system (12%) and MCA (11%). Most perinatal deaths occurred in the early neonatal period (Table 3).

**TABLE 3:** Mortality among cases with congenital anomalies according to type of anomaly (*n* = 8535, perinatal mortality in grey); Eurocat Northern Netherlands, 2001–2017

	Total cases	Early foetal mortality		Late foetal mortality		Early neonatal mortality	Total mortality
		spontaneous	TOPFA	spontaneous	TOPFA	0–7 days	
Type of anomaly	*N*	*n* (%)	*n* (%)	*n* (%)	*n* (%)	*n* (%)	*n* (%)
Structural anomalies	5746	37 (0.6)	286 (5.0)	49 (0.9)	6 (0.1)	77 (1.3)	455 (7.9)
*Central nervous*	*330*	*2 (0.6)*	*145 (43.9)*	12 (3.6)	2 (0.6)	25 (7.6)	186 (56.4)
*Cardiac and circulatory*	*1615*	*4 (0.2)*	*60 (3.7)*	16 (1.0)	1 (0.1)	26 (1.6)	*107 (6.6)*
*Digestive*	*596*	*0 (0.0)*	*0 (0.0)*	0 (0.0)	0 (0.0)	1 (0.2)	*1 (0.2)*
*Urogenital*	*1422*	*6 (0.4)*	*43 (3.0)*	11 (0.8)	2 (0.1)	13 (0.9)	*75 (5.3)*
*Orofacial clefts*	*447*	*3 (0.7)*	*5 (1.1)*	1 (0.2)	1 (0.2)	0 (0.0)	*10 (2.2)*
*Musculoskeletal*	*929*	*5 (0.5)*	*2 (0.2)*	2 (0.2)	0 (0.0)	0 (0.0)	*9 (1.0)*
*Other*	*407*	*17 (4.2)*	*31 (7.6)*	7 (1.7)	0 (0.0)	12 (2.9)	*67 (16.5)*
MCA	466	16 (3.4)	72 (15.5)	21 (4.5)	1 (0.2)	28 (6.0)	138 (29.6)
Abnormal karyotype	961	71 (7.4)	450 (46.8)	51 (5.3)	7 (0.7)	28 (2.9)	607 (63.2)
Other syndromes	1362	21 (1.5)	189 (13.9)	26 (1.9)	4 (0.3)	49 (3.6)	289 (21.2)
Total	8535	145 (1.7)	997 (11.7)	147 (1.7)	18 (0.2)	182 (2.1)	347 (17.4)

Abbreviations: MCA, multiple congenital anomalies TOPFA, elective termination of pregnancy for foetal anomalies.

*Note*: The Grey Shade terms definitions are Late foetal mortality: Foetal death (spontaneous and elective) and stillbirths at or after 24 completed weeks of gestation, Early neonatal mortality: Death in live births (at or after 24 completed weeks of gestation) until day 7 after birth, Early foetal mortality: Foetal death (spontaneous and elective) and stillbirths before 24 completed weeks of gestation.

### 3.3 Trends in mortality

**FIGURE 1:**
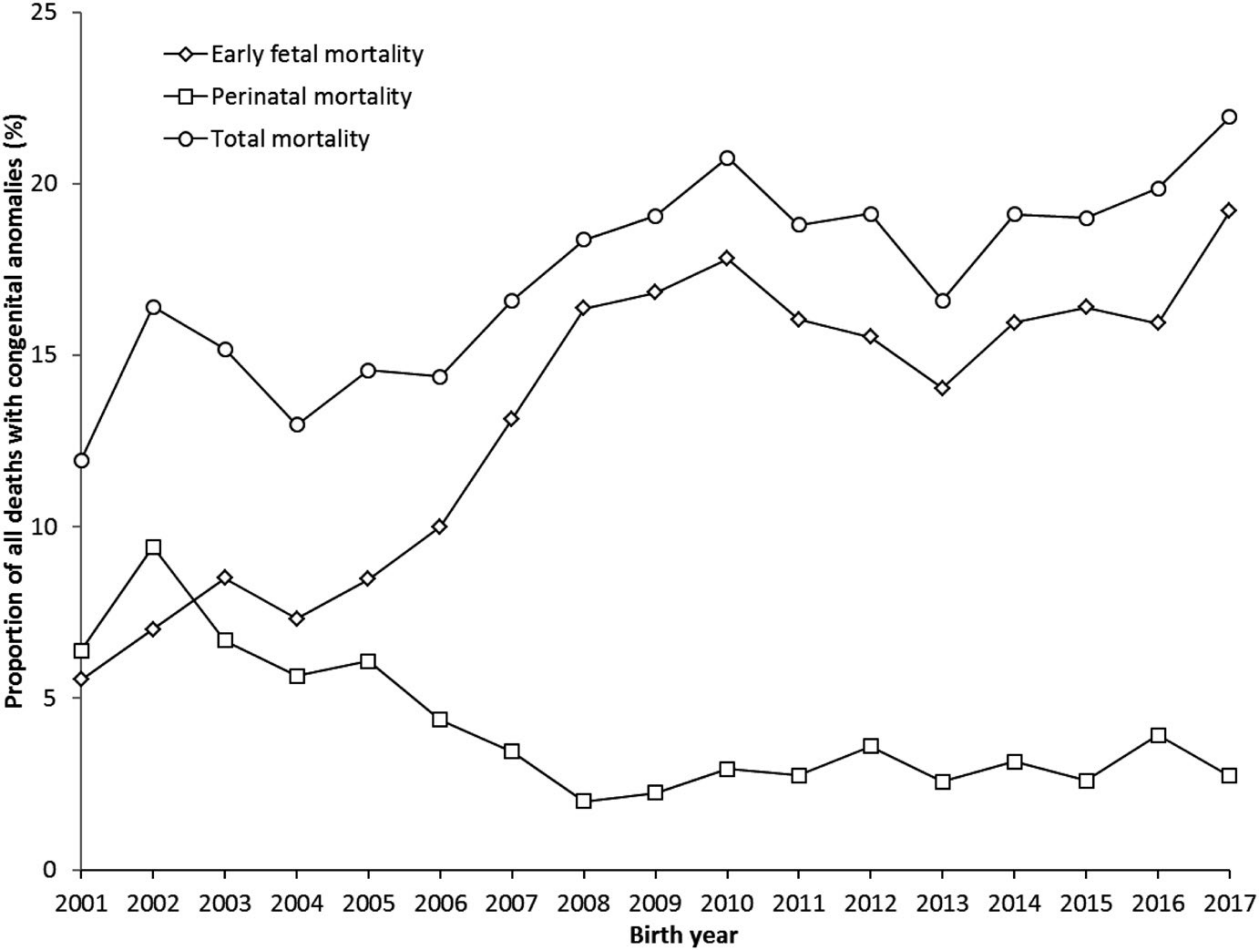
Mortality among cases with congenital anomalies (CA) per birth year (*n* = 8535); Eurocat Northern Netherlands, 2001‒2017

Between 2001 and 2017, we observed an increase in total mortality from 11.9% in 2001 to 21.9% in 2017 (AAPC 2.6, 95% CI 1.5, 3.7). This increase in total mortality was caused by an increase in early foetal mortality (5.5% in 2001 vs. 19.2% in 2017; AAPC 8.7, 95% CI 4.7, 12.9), whereas perinatal mortality decreased over the same period from 6.4% to 2.7% (AAPC −5.6, 95% CI −10.0, −1.0) (Figure 1). Joinpoint analysis showed increasing trend in early foetal mortality and decreasing trend in perinatal mortality, mainly visible in birth years 2001‒2009 (Appendix S1 and S2).

**FIGURE 2:**
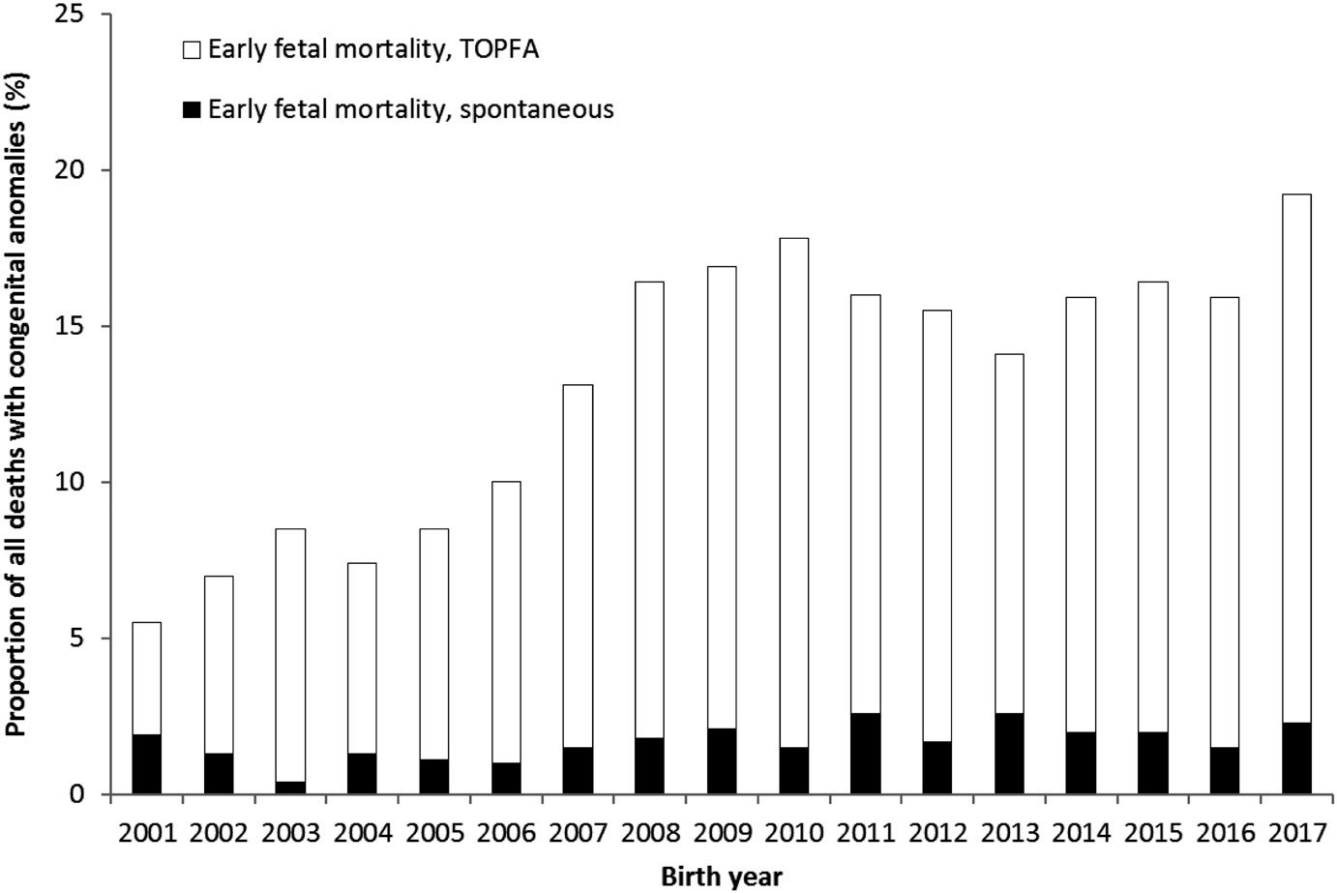
Early foetal mortality (<24 weeks’ gestation) among cases with congenital anomalies (CA) per birth year according to type of mortality; Eurocat Northern Netherlands, 2001‒2017

The increase in early foetal mortality was caused by an increase in TOPFAs from 3.6% to 16.9% (AAPC 8.3, 95% CI 4.2, 12.7) (Figure 2), although spontaneous early foetal mortality also increased slightly from 1.9% to 2.3% (AAPC 4.7, 95% CI 1.0, 7.5). Joinpoint analyses showed increasing trend in TOPFA, mainly between 2001 and 2008, and no specific periodic trends for spontaneous early foetal mortality (Appendix S3 and S4). Additionally, most cases of early foetal mortality were associated with abnormal foetal karyotype. Although early foetal mortality associated with isolated structural anomalies increased particularly after 2006 (Figure S1).

**FIGURE 3:**
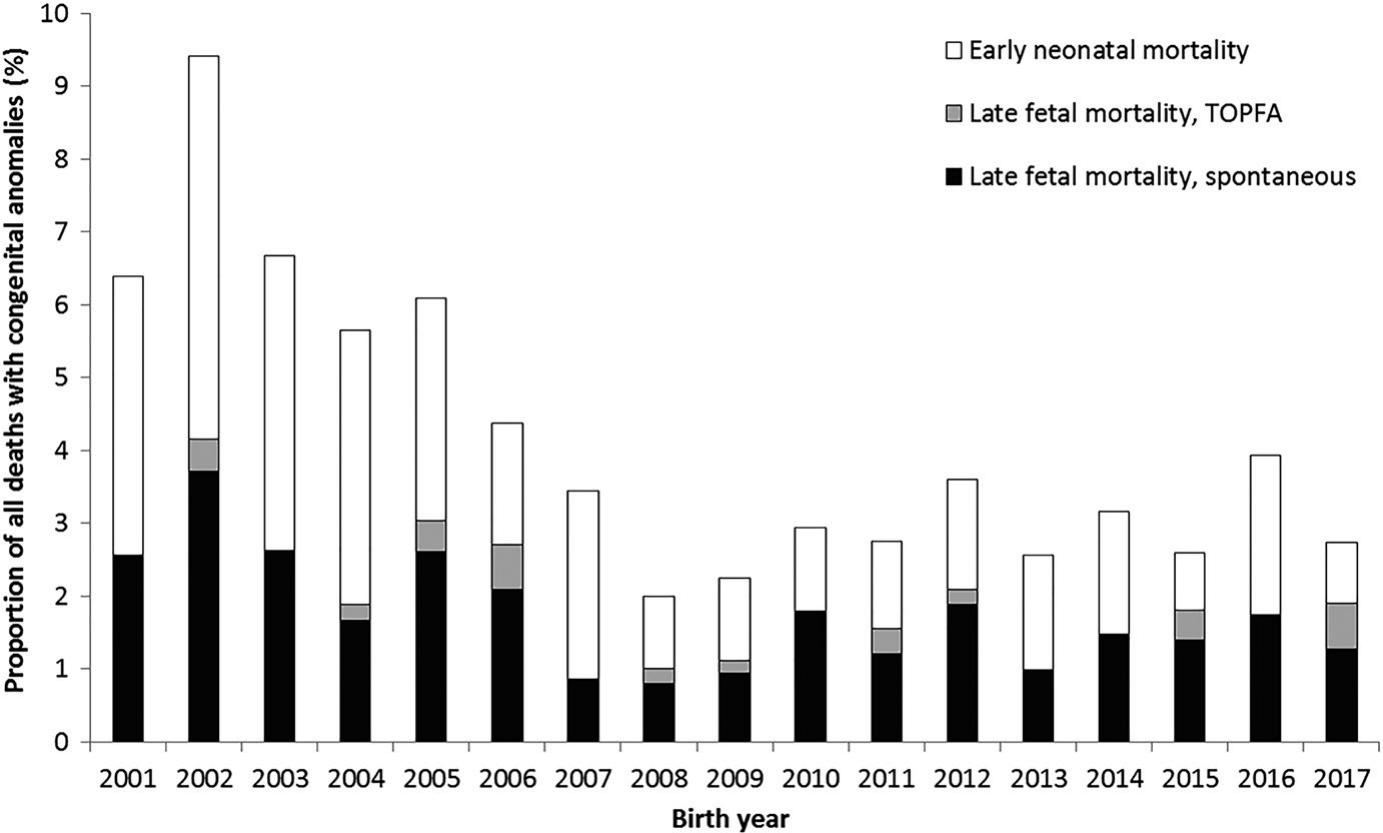
Perinatal mortality (foetal mortality ≥24 weeks and neonatal mortality ≤7 days) among cases with congenital anomalies (CA) per birth year according to type of mortality; Eurocat Northern Netherlands, 2001‒2017

Within perinatal mortality, both late foetal and neonatal mortality decreased between 2001 and 2017 (late foetal mortality dropped from 2.6% in 2001 to 1.9% in 2017 (AAPC −4.2, 95% CI −7.6, −0.7%); early neonatal mortality from 3.8% to 0.8% (AAPC −6.6, 95% CI −13.1, 0.5) (Figure 3). Joinpoint analysis found overall decreasing trends for late foetal and early neonatal mortality, but no specific periodic trends (Appendix S5 and S6). A decrease in perinatal mortality was observed in foetuses with isolated structural anomalies and abnormal karyotype (Figure S2).

Foetal mortality mostly occurred around 13‒14 and 20‒23 weeks of gestation, and the majority of these cases were TOPFAs (Figure S3a). The majority of deaths in the first trimester were observed in foetuses with abnormal karyotype, whereas second‐trimester mortality was most often associated with isolated structural anomalies (Figure S3b, c). Neonatal death mainly occurred on the day of birth or the first day post‐partum (59%, data not shown).

## 4 COMMENT

### 4.1 Principal findings

In our population‐based study on total, early foetal and perinatal mortality associated with congenital anomalies, we observed a remarkable increase in total mortality and shift in the timing of mortality between 2001 and 2017. The most evident change was a large (almost fourfold) increase in early foetal mortality, mainly caused by a remarkable rise in the proportion of early TOPFAs. Perinatal mortality in foetuses/neonates with congenital anomalies declined and the most evident decrease occurred in cases with isolated structural anomalies. Early foetal mortality was much more pronounced in foetuses with an abnormal karyotype and isolated structural anomalies.

### 4.2 Strengths of the study

A strength of the study is that it includes data on all types of birth, including early TOPFAs, making it possible to describe true total mortality rates (in contrast to limiting the numbers to stillbirths). Most previous studies have only focused on stillbirths, without reporting TOPFAs, thereby underestimating the effect of prenatal screening on early foetal mortality.^14^, ^15^ Another strength of this study is that Eurocat NNL is a population‐based registry using active case ascertainment and including all cases of congenital anomalies registered in the Northern Netherlands using a standardised methodology for coding and classification. The registry is representative of the overall population of foetuses/children with congenital anomalies in this region.

### 4.3 Limitations of the data

The lack of national data represents a limitation of the study. Moreover, it is important to note that the uptake of first‐trimester screening in the Northern Netherlands is below the national average (uptake CT: 32% NNL vs. 52% nationally), and this might have resulted in a higher proportion of TOPFAs being performed after 20 weeks of gestation following the second‐trimester anomaly scan.^16^ Additionally, early foetal mortality due to TOPFAs might be overestimated as some very early losses would not be recognised to be affected by any malformation if they just resulted in a miscarriage before first‐trimester screening is performed, whereas they are counted if prenatally diagnosed and terminated. Also, even though the Dutch prenatal screening programme has considerably changed throughout the study period, the number of cases is too small to effectively study the effects of such changes on mortality rates. In addition, the process of case registration and follow‐up collection takes time and depends on the moment of diagnosis of congenital anomalies. Indeed, milder anomalies presenting later in childhood might be underreported in the most recent study years. As a consequence, mortality rates might be overestimated for those years. Thorough monitoring is therefore essential to properly evaluate data registration quality. Finally, although we presented data on maternal educational level, we did not study the effects of deprivation on participating in the prenatal screening programme and on mortality rates.

### 4.4 Interpretation

The prevalence of congenital anomalies in Europe has been changing in the past decades and is still continuously evolving. While the reasons behind the rise in prevalence of some foetal anomalies still remain unclear, some factors have been recognised as contributors of higher rates of congenital anomalies. For instance, higher prevalence of maternal risk factors, such as obesity and diabetes, has been associated with higher rates of some heart defects. By allowing for earlier diagnosis during pregnancy, prenatal screening is an important contributor to the epidemiology of congenital anomalies.^17^ Since TOPFAs account for a non‐negligible proportion of all cases affected with congenital anomalies, substantial selection bias is likely to be present when TOPFAs are omitted in studies on risk factors or from mortality rates in evaluation studies.^18^, ^19^

The major role of prenatal screening in reshaping the distribution of foetal and neonatal deaths has been previously shown.^20^ The increasing trend in early foetal mortality and decreasing trend in perinatal mortality in the Eurocat NNL data were mainly visible in the period before and right after (2001–2009) the introduction of the national prenatal screening programme. In the years before the introduction of the national prenatal screening programme, screening for chromosomal anomalies and ultrasound examinations were performed increasingly in research settings and on maternal request, but not as part of a prenatal screening programme.^21^, ^22^ Therefore, changes are already visible before the official introduction. After implementation of the prenatal screening programme, perinatal mortality became stable. We observed a much larger number of TOPFAs being performed at lower gestational ages, as well as a fourfold decrease in early neonatal mortality in children with congenital anomalies.

The major contribution of TOPFAs to foetal and neonatal mortality figures has been described previously.^23^, ^24^ In the Eurocat NNL data, this is further supported by two clear peaks in TOPFA rates at 13–14 and at 21–23 weeks of gestation, which follow the timing of first‐trimester screening (CT/NIPT) at 11–13 weeks and second‐trimester anomaly scan at 20 weeks of gestation. A recently published study by van der Meij and colleagues showed that national first‐trimester screening uptake (CT +NIPT) increased from 15% in 2007 to 46% in 2017–2019, further supporting the rise in early TOPFAs.^25^ Some of the terminated pregnancies might have otherwise resulted in spontaneous late foetal or early neonatal deaths; therefore, explaining the lower perinatal mortality rates recorded after 2007.^20^ Indeed, countries where TOP is not allowed or only available for certain indications have the highest neonatal mortality rates for congenital anomalies.^26^, ^27^

When discussing the contribution of TOPFAs to the redistribution of mortality rates, it is essential to address the broader purposes of prenatal screening. Prenatal screening is designed to determine whether a pregnant woman is at increased risk of carrying a foetus affected by a structural or genetic anomaly and to offer her the possibility of undergoing additional diagnostic testing to obtain a definitive diagnosis.^28^ Both prenatal screening and diagnosis are on a voluntary basis and it is up to parents to decide whether they wish to opt for them. Once an anomaly is found, parents are counselled on the prognosis of their child and the therapeutic options, and are given the opportunity to make an informed decision on the management of pregnancy. The rise in total and early foetal mortality rates following the increase in early TOPFAs is therefore the result of an informed parental decision to terminate the pregnancy.

Our study demonstrates that the introduction of the prenatal screening programme in 2007 resulted in a clear increase in total mortality and shift from late foetal and neonatal to early foetal mortality. The overall perinatal mortality rate (not restricted to congenital anomalies) decreased in the Netherlands in the period in which the prenatal screening programme was implemented, from 10.5 per 1000 births in 2004 to 7.7 in 2015.^27^, ^29^ As shown in the current study, prenatal screening led to an overall increase in total mortality and a remarkable shift in timing of mortality. However, since the data used in the Euro‐Peristat report for the Netherlands could not distinguish between spontaneous foetal mortality and TOPFA, and the definition of perinatal mortality included foetal mortality ≥22 weeks of gestation, we are unable to determine the impact of the prenatal screening programme (or other improvements in prenatal care) on the national decrease in overall perinatal mortality.^30^

Next to very preterm birth and foetal growth restriction, congenital anomalies are the principal contributors to perinatal mortality.^27^ However, because of the poor prognosis of the most severe anomalies and the risk of long‐term disability, the rates of termination of pregnancy are much higher in cases with congenital anomalies.^31^ When parents opt for pregnancy continuation, delivery of affected newborns is scheduled in tertiary care centres with neonatal intensive care units where postnatal surgical correction of structural defects can take place. Indeed, neonatal surgery has been proven to be an effective strategy to considerably reduce neonatal deaths, especially in newborns with isolated abdominal wall, gastrointestinal, cardiac and diaphragmatic congenital defects.^32^ Foetal surgery for structural anomalies is a rapidly developing field that could further contribute to the reduction in mortality rates among children with congenital anomalies. However, only a very small proportion of structural defects, such as some cases of myelomeningocele, diaphragmatic hernia or obstructive uropathy, are eligible for in utero interventions, and the success rate of such procedures is still subject to debate.^33^, ^34^

Another suggested strategy to further reduce perinatal mortality is by optimising primary prevention of congenital anomalies, for instance through folic acid supplementation or by minimising environmental risk factors in the pre/periconceptional period.^1^, ^35^ Regardless of aetiology, early diagnosis of congenital anomalies by prenatal screening is of key importance. Not only because it allows parents more time to make a well‐informed decision about termination or continuation of pregnancy but also because it offers healthcare professionals’ essential information for optimising postnatal care.

## 5 CONCLUSION

The current study demonstrates the striking influence of prenatal screening on total mortality and the pattern of foetal and neonatal mortality rates. Earlier detection of congenital anomalies resulted in a decrease in early neonatal deaths and a marked increase in early foetal deaths, which is explained by a much higher proportion of TOPFAs. Given the often‐poor prognosis of severe congenital anomalies, risk stratification during pregnancy and extensive parental counselling are necessary steps towards the optimisation of postnatal care in affected children.

## Supporting information

Figure S1

Figure S2

Figure S3

Appendix S1

Appendix S2

Appendix S3

Appendix S4

Appendix S5

Appendix S6
