# Supplementary material for: Effect of prenatal screening on trends in perinatal mortality associated with congenital anomalies before and after the introduction of prenatal screening: A population‐based study in the Northern Netherlands
Source: Paediatr Perinat Epidemiol. 2021 Jul 30;35(6):654–63. doi: 10.1111/ppe.12792 (PMC8596841; doi:10.1111/ppe.12792)

***eFigure 1****. Early fetal mortality (<24 weeks’ gestation) among cases with congenital anomalies (CA) per birth year, according to type of congenital anomaly); Eurocat Northern Netherlands, 2001–2017.*


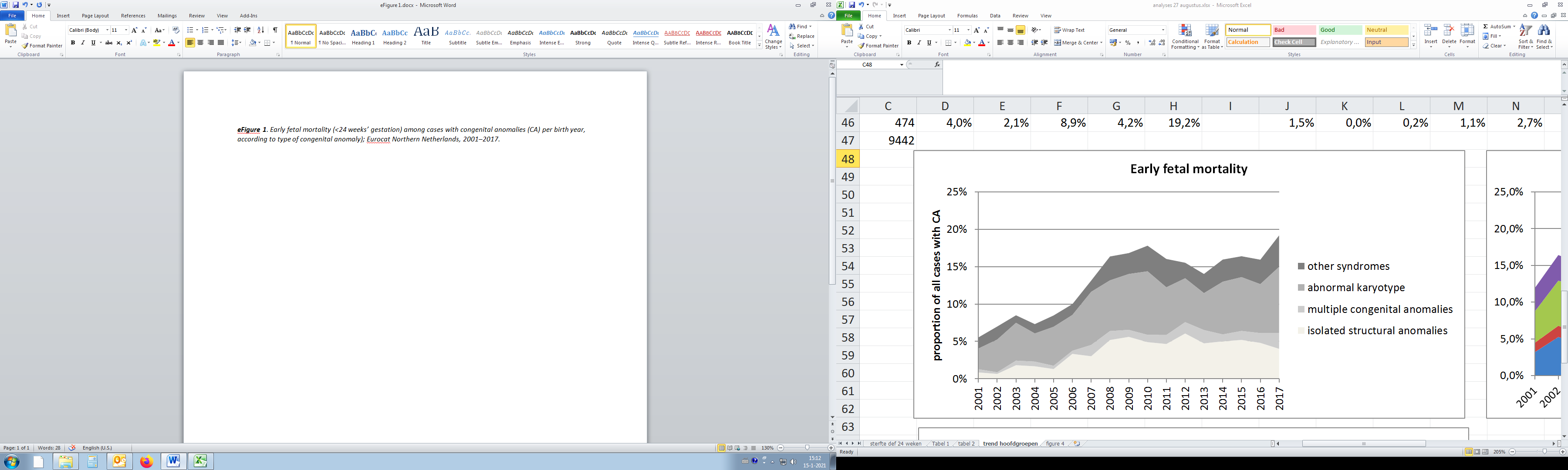

Supplement: Supplementary file 1 — Figure S1 [file PPE-35-654-s005.docx]
