# Supplementary material for: Effect of prenatal screening on trends in perinatal mortality associated with congenital anomalies before and after the introduction of prenatal screening: A population‐based study in the Northern Netherlands
Source: Paediatr Perinat Epidemiol. 2021 Jul 30;35(6):654–63. doi: 10.1111/ppe.12792 (PMC8596841; doi:10.1111/ppe.12792)

***eFigure 2****. Perinatal mortality (fetal mortality ≥24 weeks and neonatal mortality ≤7 days) among cases with congenital anomalies (CA) per birth year, according to type of congenital anomaly); Eurocat Northern Netherlands 2001–2017.*


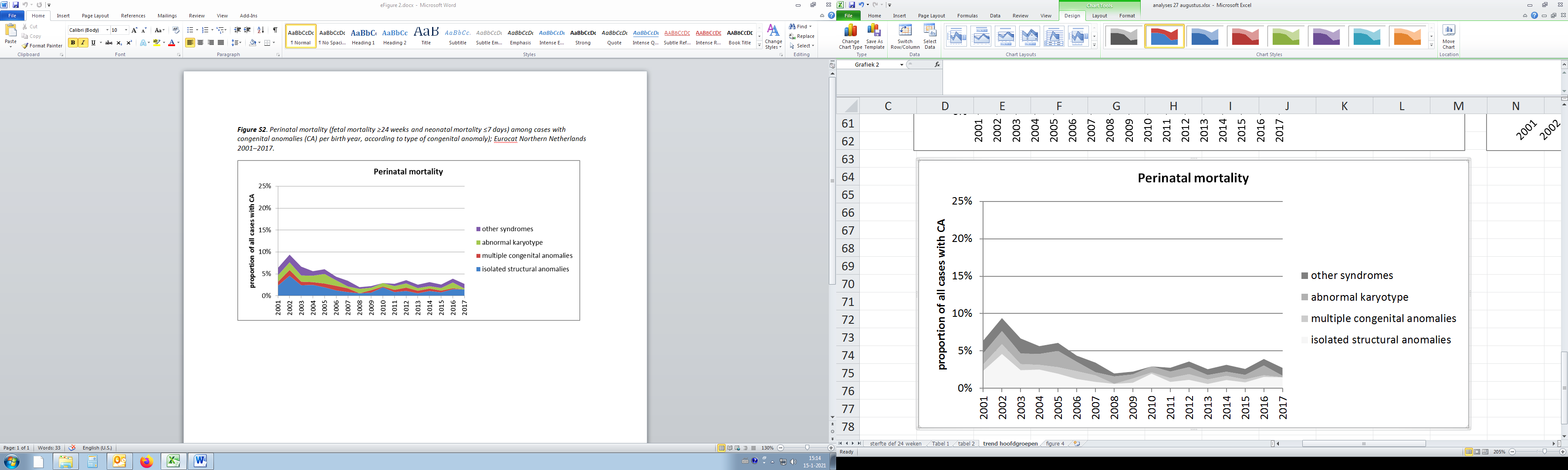

Supplement: Supplementary file 2 — Figure S2 [file PPE-35-654-s009.docx]
