# Supplementary material for: Effect of prenatal screening on trends in perinatal mortality associated with congenital anomalies before and after the introduction of prenatal screening: A population‐based study in the Northern Netherlands
Source: Paediatr Perinat Epidemiol. 2021 Jul 30;35(6):654–63. doi: 10.1111/ppe.12792 (PMC8596841; doi:10.1111/ppe.12792)
Supplement: Supplementary file 3 — Figure S3 [file PPE-35-654-s008.docx]

***eFigure 3.*** *Distribution of the fetal mortality at gestational age according to type of mortality (spontaneous fetal death and termination of pregnancy) for all anomalies (3a), isolated anomalies (3b) and abnormal karyotype (3c). Total fetal mortality is 100% (n=1299 cases). Eurocat Northern Netherlands 2001-2017.*
