# Supplementary material for: Effect of prenatal screening on trends in perinatal mortality associated with congenital anomalies before and after the introduction of prenatal screening: A population‐based study in the Northern Netherlands
Source: Paediatr Perinat Epidemiol. 2021 Jul 30;35(6):654–63. doi: 10.1111/ppe.12792 (PMC8596841; doi:10.1111/ppe.12792)
Supplement: Supplementary file 6 — Appendix S3 [file PPE-35-654-s006.docx]

**Supplementary information S3**. Trends in early fetal mortality through TOPFA among cases with congenital anomalies, using Joinpoint regression, Eurocat Northern Netherlands, 2001‒2017.
